# Factors Affecting Brain Drain and a Solution to Reduce it in Iran’s Health System: A Qualitative Study

**DOI:** 10.34172/aim.28863

**Published:** 2024-08-01

**Authors:** Mohadeseh Ghanbari-Jahromi, Milad Ahmadi Marzaleh

**Affiliations:** ^1^Research Center for Social Determinants of Health, Jahrom University of Medical Sciences, Jahrom, Iran; ^2^Department of Health in Disasters and Emergencies, School of Health Management and Information Sciences, Shiraz University of Medical Sciences, Shiraz, Iran

**Keywords:** Brain drain, Developing countries, Health, Human resources, Immigration, University

## Abstract

**Background:**

Brain drain is an issue of concern in developing countries. Many factors are involved in this issue, and their identification can be a good guide for decision-makers at different management levels. Therefore, the present study was carried out to identify the factors affecting brain drain and solutions to reduce it in Iran’s health system.

**Methods:**

The data for this qualitative study was collected in 2023 through 15 semi-structured interviews with Iranian health experts and emigrant elites. Interviews were collected both face-to-face and virtually (on Skype and Google Meet) and analyzed using the thematic content analysis method.

**Results:**

Data analysis of the factors affecting brain drain in Iran’s health system yielded seven main categories and 45 subcategories. The main categories were "individual factors," "economic factors," "social and cultural factors," "organizational and administrative factors," "political factors," "attraction factors for destination countries," and "attraction factors of destination countries for elites." Also, the solutions were divided into four categories of "economic," "social," "organizational and scientific," and "political" factors and 19 subcategories.

**Conclusion:**

Due to the increase in brain drain in recent years, it is necessary to make a serious decision in dealing with this issue. Solutions such as creating stable economic conditions, creating an atmosphere of hope, increasing respect and dignity for elite students by politicians, using the elites in macro-decisions, and creating the job rank for honorary professors can lead to reducing brain drain and decreasing the costs of the health system.

## Introduction

 Universal health coverage is one of the main goals of health systems, which requires sufficient, skilled, and motivated health workers.^1^ In recent years, the shortage and inappropriate distribution of healthcare workers have reached a critical stage.^2^ The World Health Organization (WHO) has predicted that this shortage will reach 15 million people by 2030.^3,4^ Also, according to WHO estimates, there is a shortage of 2.8 million doctors in developing countries due to migration.^5^ In low- and middle-income countries (LMICs), the need for health professionals is more intense because these countries are unable to provide the minimum initial threshold of 23 health professionals per 10 000 population.^3^ These shortages worsen with the migration of educated and skilled health workers from LMICs to high-income countries (HICs). The negative impact of these migrations is more felt in low-income countries.^6^ Loss of trained health personnel in the health systems demoralizes the remaining professionals. This aggravates the low doctor-patient ratio and causes a lack of expert staff and a decrease in the quality of care provided.^7,8^

 The migration of skilled workers from LMICs to HICs, so-called brain drain,^9^ is a very worrying issue for the health system of LMICs. The migration of elites and professionals to developed countries is another challenging issue that has significant consequences for the country of origin.^10^ An elite is a person who plays an effective role in the historical and social activities of societies by using innovative ideas and fateful decisions.^11^ The exodus of educated people, known as elite migration or, more pessimistically, brain drain, has increased greatly in recent years.^12^ The number of doctors and nurses emigrating to OECD countries has increased by 61% from 2010 to 2020.^13^ According to the statistics of Iran’s Migration Observatory in 2022, the number of Iranian students studying in foreign countries has increased from 19 000 in 2003 to 56 000 in 2018, and the number of Iranian immigrants in 1990 was 820 000 which reached 1.8 million in 2020. This shows an increase of 2.2 folds in the number of Iranian emigrants in the last 30 years.^14^ According to unofficial statistics, the emigration of specialists in the last three years has reached 4000 doctors, and that of those with master’s and doctorate degrees has reached 300 000 people. The emigration of elites leads to the loss of profits for the country of origin, and the receipt of profits for the destination country due to the presence and use of the knowledge of these elites.^15^

 In general, brain drain in LMICs occurs for various reasons. In these countries, talented people do not return to their country of origin due to the lack of favorable opportunities in various fields of science, technology, and medicine and issues related to better income and better opportunities for professional development.^16^ According to a study by Malekpour Afshar et al, economic, social, organizational, administrative, and political factors are among the reasons for elite emigration.^17^ Heidary Soureshjani and Falahaty found that the most important reasons for the elites’ tendency to emigrate were social welfare, job satisfaction, freedom of expression, high research opportunities and facilities, and the absence of burdensome laws for the elites in the destination country.^18^ According to the study by Pieńkowski, financial markets, insufficient pensions, and insurance in Ukraine are among the reasons for labor migration.^19^ The study by Yakovlev and Steinkopf on the physicians’ brain drain from 144 countries of origin to 18 countries of destination showed that doctors are more attracted to countries with more economic freedom and those with a lower share of public health costs and per capita health costs.^20^ The study of Balouch et al in Pakistan showed that the low quality of education and poor working environment were among the reasons for the departure of doctors from Pakistan, and the United Kingdom was the most popular destination for doctors.^21^ According to the study by Kabbash et al, Egyptian medical students migrate to seek better work and financial incentives. These doctors admitted that only 4.9% of them felt appreciated by the country; 75.9% were not satisfied with their relationship with patients, and 40.2% were not satisfied with their relationship with their colleagues. Verbal abuse was reported by 55.5% of students, and physical assaults by 35.4%. According to the opinion of the majority of medical students who intended to emigrate (85.1%), if there are improvements in the health sector, they will change their opinion.^22^

 Therefore, due to the importance of the issue of brain drain and the irreparable consequences it has on the future of the country, the present study was conducted with the aim of investigating the factors affecting brain drain and ways to reduce it in Iran’s health system in 2023.

## Materials and Methods

###  Participants

 The participants were divided into two categories: health system experts in different fields and emigrant elites. They were selected using purposive and snowball sampling with the maximum diversity approach to obtain a comprehensive view of the participants on the subject under investigation. Interviews were conducted until data saturation was reached and no new results were obtained any more. Finally, 11 health system experts in different fields and 4 elite people who emigrated from Iran to other countries were selected for interviews. To ensure anonymity, each participant was assigned a unique code.

###  Data Collection

 A qualitative approach was used for this research, and the data were collected through semi-structured interviews. For this purpose, experts were asked questions such as “1. What are the factors affecting the occurrence of brain drain in Iran’s health system?” and “2. What are the solutions to reduce the occurrence of brain drain in Iran?” However, during the interviews and according to the answers of the participants, further questions were asked. Accordingly, in-depth and semi-structured interviews were conducted from May 1 to September 1, 2023, with health system experts and elites who had emigrated.

 Interviews were conducted face-to-face and virtually (on Skype and Google Meet). The interviews lasted from 30 to 60 minutes, and the average time was 45 minutes. The average time for face-to-face interviews was 50 minutes. The interviews were recorded and transcribed verbatim. Coding was reviewed and confirmed by two experts in qualitative research.

###  Data Analysis

 Data analysis was done manually. To extract the concepts from Brown and Clark’s thematic analysis method, we performed data analysis in six steps.^23^ At first, the text of the interviews was re-read and coded several times so that in the first stage (familiarity with the data), the researcher repeated the re-reading of the interviews to immerse himself in the study data to get the general ideas of the interview. In the second stage (production of primary codes), meaning units were extracted from the text of the interviews and coded primarily. The third stage (searching within the themes) was carried out to compile the themes from the primary codes. This means that similar codes were classified into a class under the title of the topic. In the fourth stage (revision of the subjects), considering the internal and external similarity, we tried to distinguish the data in each subject in addition to the internal continuity with the data in other subjects. In the fifth stage (defining and numbering of the elements), which was done to compile a classification, the essences of each element were identified, and in the final stage, an attempt was made to gather all the meanings of the elements to give a general expression of the factors affecting brain drain and its reduction strategies in Iran’s health system.

###  Trustworthiness and Rigor

 The reliability of the data was evaluated according to the criteria proposed by Guba and Lincoln, i.e. validity, reliability, transferability, and confirmation.^24^ Validity criteria were achieved through extended interaction with the data, member checking during the data analysis process, and peer review as an external check of the data. Reliability measurement was performed by researchers who documented the research process in detail. A detailed description of the study process was provided to confirm the transferability. To increase verifiability, two external experts familiar with qualitative research methods were requested to verify the accuracy of the study process and data coding.

## Results

 The median (IQR: Q1, Q3) age and work experience of 15 participants in this research was 40 (36,48) and 10 (5,15) years, respectively. Also, 93% of them were male. The demographic features are presented in Table 1.

**Table 1:** Demographic Characteristics of the Participants

**Variables**		**Participant (n=15)**
Age, year, median (IQR: Q1, Q3)		40 (36,48)
Work experience, year, median (IQR: Q1, Q3)		10 (5,15)
Gender, No. (%)	Male	14 (93)
	Female	1 (7)
Education level, No. (%)	MSc	1 (7)
	MD	4(27)
	Pharm	2(13)
	Ph.D.	8(53)
Field, No. (%)	Health Policy	1(7)
	Medical Physics	1(7)
	Health in disasters and emergencies	2(13)
	Cell therapy	1(7)
	Social medicine	1(7)
	Public health	1(7)
	Proteomics	1(7)
	Immunology	1(7)
	Pharmacology	2(13)
	Internal medicine	1(7)
	Epidemiology	1(7)
	General practitioner	2(13)
Migration, No. (%)	Migration	4(27)
	No immigration	11(73)

 After analyzing and interpreting the data, a total of 305 codes were extracted, and after removal of duplicate codes and merging of similar ones, 45 were finally left. Seven main categories, including individual, economic, social, cultural, organizational, administrative, scientific, and political factors, as well as attraction factors for destination countries, and attraction factors of destination countries for elites, were identified. Each of these main categories encompasses several subcategories. The thematic framework of the findings is presented in Table 2.

**Table 2:** Factors Affecting Brain Drain in Iran's Health System

**Category**	**Subcategory**	**Number of Participants**
Individual factors	Individual's lack of adaptation to the conditions of the country	5
	Comparison of their conditions with other people in other countries	5
	Despair about the future	1,3,5,6,8,9,10,11
	Decrease in the motivation of elite students and professors	2,7,8
	Lack of mental and emotional peace	4,5,7
	Lack of proper respect for the elites	5,6,7,8
Economic factors	Economic instability	1,4,5,6,10,12,14
	Low economic growth	2
	High inflation	3,6,7,8,9
	Economical pressure	4
	Ignorance of the financial needs of elites	4,8
	Lack of suitable labor market	3,5,6,11
	High taxes	1,2,7
	Inequality of people in salaries and wages	1,6,8,12
Social and cultural factors	Many social constraints	1
	Inducing the thoughts of friends, family, and acquaintances	1,5,7
	Bad effects of virtual space	1,5,7,11
	Uncertain future of children	1,5,8,10,12
	People's anonymity	2
	Luxury	5,7
	Existence of social tensions and conflicts	1,6,10,12,14
	Cultural transformation	1,3,6
Organizational and administrative factors	High work pressure	1,10
	Lack of meritocracy	3,5,6,7,9,12,13,15
	The existence of brokers and organizational mafia	4,7
	Many organizational constraints	1
	Ethnic discrimination	3
Political factors	Distrust of government policies	1,5,8
	Increase in the capacity of medical groups	1,2,3,4,5,7,8,9,10
	Low international communication	2,12,15
	Insufficient attention to elites	2,4,7
	Political game by destination countries	3
	Changing government policies	4,5,6,7,9
	lack of using knowledgeable advisors in making decisions	8,9
	Challenges of technology and innovation	6
	Little efforts by politicians to change the situation	1,3,6,7,12,15
	Lack of acceptance of constructive criticism by managers and politicians	8
	Existence of excessive academic and employment quotas	2
	Malicious advertising of destination countries	1
	Lack of research opportunities	3,4
Attraction factors for destination countries	Compensation for lack of population	2,4,5
	Interest in collecting elite genetic stocks from other countries	8
Attraction factors of destination countries for elites	Predictability of the economy	1,3,4,9
	Better quality of life	12
	High salary	12

###  Individual Factors

 Subcategories related to individual factors include the individual’s lack of adaptation to the conditions of the country, comparison of their conditions with other people in other countries, despair about the future, decrease in the motivation of elite students and professors, lack of mental and emotional peace, and lack of proper respect for the elites.

 In this part, many of the interviewees pointed to the despair about the future as one of the most important factors in brain drain.

 “*My life is based on not getting worse, but the people of other countries hope to get better! I have no hope; I’m just trying to get through my affairs...” (*P1, Health Policy).

 “*The hopes of the people who were hoping and staying in the country have diminished. Then, they say come and spread hope in the society. You created the conditions in such a way that the professors who stayed and instilled hope in the students, now don’t have any hope either!” *(P8, Immunology).

 From the participants’ point of view, lack of proper respect for the elites and lack of attention to their demands are one of the reasons for the departure of the elites from the country. The interviewees acknowledged:

 “*I think many of the fields that are not part of the triangle fields (medicine, dentistry, and pharmacy) are constantly insulted and humiliated and are looked at inferiorly. This greatly affects their progress!” *(P7, Elite immigrant, Public health).

 “*During the time of COVID-19, the issue of the COVID-19 reward was raised to appreciate the efforts of nurses, but it was so little that it would have been much better if they had not been given! In fact, they humiliated nurses by doing this…” *(P6, Elite immigrant, Proteomics).

###  Economic Factors

 One of the most effective factors in brain drain is the economic factor, which includes economic instability, low economic growth, high inflation, economic pressure, ignorance of the financial needs of elites, lack of a suitable labor market, high taxes, and inequality of people in salaries and wages. In the meantime, economic instability, high inflation, and inequality of salaries and wages among people are among the most important factors affecting brain drain.

 From the participants’ point of view, economic instability is a very important factor in the departure of elites. Two of the participants stated:

 “*My field is laboratory sciences; financial issues and economic instability in the country have disrupted all our plans. Most of our equipment is imported. Every time the rate of dollar rises, the price of laboratory equipment increases terribly or becomes scarce. In the meantime, the professor does not dare to pay for the student’s project because it is not known when the university will return the money, and when it is returned, it will not have the same value as before. Because of this instability, many projects are left unfinished...” *(P4, Elite immigrant, Cell therapy).

 “*In the current situation, I think the main reason for the brain drain is economic instability. Considering the current crises and sanctions, there is no certainty that the situation will improve or not. That’s why the brain drain has intensified...” *(P6, Elite immigrant, Proteomics).

 High inflation was one of the effective factors in the departure of elites from the participant’s point of view. For example, the participants stated:

 “* Laws are not enforced properly. Inflation is so high that a person cannot plan for his/her life. How can a young man who just wants to start his life cope with this inflation? For example, house rent used to increase by a small percentage per year, but now it increases several times within a few months, not only the house rent but also food and clothing. This high price and high inflation are back-breaking...” *(P7, Elite immigrant, Public health).

 “*After working for several years, I still cannot buy a house. I had saved money to buy it this year, but the inflation went up so much that I didn’t have any hope of being able to own a house. Then, how can a newly graduated young man own a house?! If he can afford the normal expenses of his life, he has done a great job. This is what encourages the young man to leave the country. At least, there he earns money in dollars and spends in dollars, too! Here, your whole life is in dollars, but your money is Rials...” *(P9, Pharmacology).

 Another effective factor in brain drain is the inequality of people in salaries and wages. A participant said:

 “*We have a lot of class differences in the payment of salaries. There is a ceiling for salaries, which means that if you work more than the ceiling, you will not be paid. One of the professors was like this, and he was not paid his salary, but the employees of the oil company protested a lot, and then they raised the salary ceiling! Now, I am a professor whose scientific base in this country is not valued at all...” (P8, Immunology).*

###  Social and Cultural Factors

 Social and cultural factors are among the other effective factors in brain drain. Many social constraints inducing the thoughts of friends, family, and acquaintances; bad effects of virtual space; uncertain future of children; people’s anonymity and luxury; existence of social tensions and conflicts; and cultural transformation are among its subclasses.

 From the participants’ point of view, the existence of social tensions and conflicts is one of the reasons for leaving the country. Some of the participants expressed their opinions as follows:

 “*With the conditions that have been created, social tensions have increased. People are looking for more social freedom and psychological security and prefer to live in a society that is socially peaceful and far from discrimination...” (P6, Elite immigrant, Proteomics).*

 “*Every person likes to live in a place where there are no social restrictions. They like to be socially comfortable and respond properly to the tensions that arise; they should listen to people’s criticisms, not be indifferent, and do nothing to improve the situation....” (P1, Health Policy).*

 The uncertain future of children was one of the most important concerns of the participants, and according to them, one of the reasons for the departure of the elites was the future of their children.

 “*In my opinion, many people leave for their children’s future because they believe that the child would be less stressed about his future and they can easily make a plan for his life and they value his knowledge...” (P5, Social medicine).*

###  Organizational and Administrative Factors

 Organizational and administrative subcategories include high work pressure, lack of meritocracy, the existence of brokers and organizational mafia, many organizational constraints, and ethnic discrimination.

 Lack of meritocracy at management levels is one of the most important issues in the third-world countries. The opinions of the participants were as follows:

 “*There is not much meritocracy here. A person with an experimental diploma is hired, but the elite who are looking for work are not hired. When the elite sees that there is no work for them in their own country, they prefer to leave!” (P13, General practitioner).*

 “*Management fields that deal with administrative issues have a lot of nepotism. Organizations operate based on nepotism. If you don’t have it, you will be denied and someone else will come. That’s what makes that elite stop working in government centers!” (P3, Health in disasters and emergencies).*

###  Political Factors

 Distrust of government policies, increase in the capacity of medical groups, poor international communication, insufficient attention to elites, political games by destination countries, changing government policies, not using knowledgeable advisors in decision-making, challenges to technology and innovation, little effort by politicians to change the situation, lack of acceptance of constructive criticism by managers and politicians, the existence of excessive academic and employment quotas, malicious advertising of destination countries, and lack of research opportunities are among the most important sub-issues in the category of political factors.

 From the interviewees’ point of view, one of the most frequent factors in brain drain was the little effort by politicians to change the situation. A number of participants mentioned this:

 “*It seems that there is no necessary courage at the management levels to reduce the brain drain. In fact, they know and understand the damage, but they don’t have the courage to express it and solve it. In other words, the problem will not be solved soon unless the upper layers of management want it...” *(P13, Epidemiology).

 “*Some things are the legal and religious rights of all people, but they are not taken into consideration.*

*They left everything. If we proceed like this economically, politically, etc., in the near future, there will be no more expert forces in the country...” *(P7, Elite immigrant, Public health).

 Changing government policies is one of the other influencing factors in brain drain. The participants stated:

 “*Changing their laws and policies is 100% effective. If there are dry and shrinking policies and they say, “we are ready to get a passport, and those who want to leave Iran faster, and if they go, we will bring experts from other countries, then it is clear.” That elite will not stay here...” *(P5, Social medicine).

 “*Yes, it definitely has an effect. When government policies change, students and professors hope for their correct decisions. How much attention do they pay to research, science, and the quality of education? But when they see that everything has become empty words, no one is willing to stay...” *(P6, Elite immigrant, Proteomics).

 Another effective factor in brain drain is lack of research opportunities. The participants said:

 “*There is little research opportunity in the country. There are very few facilities. New devices are not imported due to sanctions and economic conditions. Well, these things make the quality of research work go down...” *(P3, Health in disasters and emergencies).

 “*Scientific research is important for every country, but the grants they give us do not cover even one step of our work. Support should be such that a researcher does not have to worry about the research work at all, rather than being forced to take it easy to finish it quickly. All this is unfortunately affected by the economy...” *(P4, Elite immigrant, Cell therapy).

###  Attraction Factors for Destination Countries

 Subcategories of attraction factors for destination countries included compensation for the lack of population and interest in collecting elite genetic stocks from other countries.

 For many countries, one of the reasons for attracting immigrants is to compensate for the lack of population. One of the participants said:

 “*Destination countries are thinking about their national interests; because their population is aging, they are looking to attract good and elite people from other countries. I think it is a very good thing. We should also learn and accept elite immigrants from good countries...” *(P2, Medical Physics).

 “*One of the reasons why destination countries seek to attract elites is that their population is aging or they have a shortage of skilled workers in a field such as nursing. Thus, it accepts the amount it needs from other countries...” *(P4, Elite immigrant, Cell therapy).

###  Attraction Factors of Destination Countries for Elites

 The predictability of the economy, better quality of life, and high salary were among the subcategories of this factor. One of the most important and interesting topics for elite immigrants is the predictability of the economy in the destination country, which has a great impact on their decision to leave.

 “*In other countries, you can plan your life very easily because everything is based on its own principles and rules. Inflation and economic instability are very low, and you can save money with the money you have and get what you want like buying a house, a car, etc. very soon. In fact, a young man can make a plan, with the amount of salary I have, I can own these things in a few years...” *(P3, Health in disasters and emergencies).

###  Solutions

 In this part, after analyzing and interpreting the data, a total of 81 codes were extracted, and after removing duplicate codes and merging similar ones, 19 codes remained.

 From the perspective of the participants, the solutions were divided into four categories: economic, social, organizational, administrative, scientific, and political factors. The findings are shown in Table 3.

**Table 3:** Strategies to Reduce Brain Drain in Iran's Health System

**Category**	**Subcategory**	**Number of Participants**
Economic factors	Creating stable economic conditions in the country	3,4
	Creating financial transparency	7
Social and cultural factors	Creating an atmosphere of hope in the country	5
Organizational and scientific factors	Creating research opportunities	3,8
	Creation of meritocracy	4,5,7
	Increasing respect and dignity for elite students by politicians	5,6,9,11
	Forming a think tank with the cooperation of elite students	5
	Providing conditions for the scientific exchange of professors and students with international universities	5
	Holding scientific tours from other countries	5
Political factors	Changing the government policy	2,4
	Using elites in macro-decisions	3,5,8
	Providing encouraging, supportive, and persuasive conditions by policymakers	5
	Dealing seriously with economic brokers	7
	Giving importance to the criticism of experts	8
	Providing conditions for the entry of elites from developed countries	9,12
	Performing social engineering (training students based on needs and creating jobs)	11
	Encouraging the immigrant elites to participate in domestic projects for knowledge and technology transfer	12
	Creating the job rank for an honorary professor to use the power of the elites	12,14
	Strengthening international relations with other countries	15

###  Economic Factors

 From the point of view of the participants, important solutions in the economic field include creating stable economic conditions in the country and creating financial transparency.

 One of the most important and effective ways to reduce the departure of elites is to create stable economic conditions in the country. A participant stated:

 “*One of the reasons that makes a person think of leaving is the bad economic conditions. Be sure that if the situation improves and a young person is provided for in his life needs and does not have to worry about the future, he will return. Of course, there are other factors involved, but the economy and money are some of the important factors...” *(P3, Health in disasters and emergencies).

###  Social Factors

 Creating an atmosphere of hope and encouraging the active forces and elites of society are necessary for the progress of any country. One of the participants expressed his opinion as follows:

 “*We must provide conditions for a peaceful social atmosphere with sparks of hope. This is how we can keep our elite...” *(P5, Social medicine).

###  Organizational and Scientific Factors

 Organizational and scientific factors include the subcategories of creating research opportunities, creating a meritocracy, increasing respect and dignity for elite students by politicians, forming a think tank with the cooperation of elite students, providing conditions for the scientific exchange between professors and students with international universities, and holding scientific tours from other countries.

 Increasing respect and dignity for elite students by politicians is a strong incentive to reduce brain drain from the perspective of participants. A participant said:

 “*If our elites know that they are valued and treated properly, their needs are met and the conditions are provided for them to take steps to meet their comprehensive needs; then, we will value and respect them. If we do this, our elites will feel they have value and remain...” *(P9, Pharmacology).

###  Political Factors

 Political factors include subcategories of changing the government policy, using elites in macro-decisions, providing encouraging, supportive, and persuasive conditions by policymakers; dealing seriously with economic brokers; giving importance to the criticism of experts; providing conditions for the entry of elites from developed countries; performing social engineering (training students based on needs and creating jobs), encouraging the immigrant elites to participate in domestic projects for knowledge and technology transfer; creating the job rank for an honorary professor to use the power of the elites, and strengthening international relations with other countries.

 One of the most practical and effective solutions is to use the elites in macro-decisions of the country. One of the participants admitted:

 “*We have to move in the direction of using our elites in big decisions more. The reason for the rapid development of any society is the use of elites and their abilities. When an elite knows that he/she is used and his/her knowledge is valued, why should he/she leave?” *(P8, Immunology).

 In the current situation, the creation of the job rank of honorary professor to use the power of the elites who have migrated can be an effective solution for the return of the elites and to motivate them. The opinion of one of the participants was as follows:

 “*Using the elites who are leaving, I think, is the best solution. Many of them like to serve their country and work for a month or two when they return. But the conditions for this work must be provided at the macro level...” *(P14, General practitioner).

## Discussion

 Brain drain is an important issue in most low- and middle-income countries such as Iran. This issue will have an irreparable impact on social capital and the future of countries. Therefore, identifying the factors affecting brain drain and using the proposed solutions can be useful. The present study was conducted to determine the factors affecting brain drain and solutions to reduce it in Iran’s health system.

 The results of the study showed that “individual, economic,” “social and cultural,” “organizational and administrative” and “political” factors; attraction factors for destination countries; and attraction factors of destination countries for elites have a great impact on brain drain. Also, in the solutions section, “economic factors,” “social and cultural,” “organizational and scientific,” and “political” factors can lead to the reduction of the elites’ exit from the country.

 Despair about the future was one of the effective factors in brain drain. Malekpour Afshar et al showed that the feeling of hopelessness and despair in Iran is one of the reasons for migration.^17^ According to a study conducted by Ryazantsev et al, lack of hope for the future increases brain drains. In this situation, the elites feel that they can achieve their human rights by emigrating.^25^ It seems that despair about the future of the country is one of the most important challenges in most countries with high brain drain statistics. Finding the root of this issue, which is a combination of economic, social, and political problems, and finding practical solutions for it should be of the priority plans and decisions of the governments.

 One of the important factors at the individual level was lack of proper respect for the elites. Abou Hashish and Ashour found in their study that nurses were not recognized for their work or were not sufficiently appreciated. They also suffer from the power difference between doctors and nurses. They relate this to the bad image of nursing and the public misconception of this profession, which affected their family relationships and social life.^26^ Another study in Egypt and Jordan confirmed that the nursing profession suffers from a negative public image in the Arab world. The media perpetuate the stereotype that nurses are angels of mercy and maidservants of doctors.^27^ According to a study, one of the important reasons for brain drain in LMICs is lack of support for health professionals.^28^ A study showed that highly educated people sought to immigrate to Western countries due to lack of respect in Iran.^25^ It seems that honoring, giving a sense of importance to the elites, and not discriminating between different disciplines are some of the most important factors in preventing them from leaving. Because the motivation and feeling of being useful to the elites can provide the conditions for their professional and academic progress.

 Economic instability can be recognized as one of the most important factors in brain drain. According to the study by Derakhshan Shahrabad et al, one of the reasons for the migration of Iranian elites to other countries is the existence of economic problems.^29^ The study by Obani and Odalonu showed that the weak economic conditions and high inflation in Nigeria have led to the migration of trained and untrained professionals, including academics, doctors, nurses, engineers, and students, to developed countries.^30^ Also, Osigbesan found that bad economic conditions were one of the reasons for migration to other countries, and participants sought to solve their deficiencies in other countries.^31^ According to a study, in countries where there is economic inflation, people have fewer job opportunities and consequently less financial turnover,^32^ which can lead to leaving the country. Another study showed that high inflation and lack of job opportunities were among the factors contributing to migration abroad.^33^ Inappropriate economic conditions and inflation have a very irreparable impact on the lives of all members of society, especially educated people. Due to the devaluation of the national currency, many of the basic needs of the elite are not being met. Therefore, economic stability is one of the important pulling factors for leaving the country, and this is why the statesmen should look for a long-term solution to solve it faster.

 Inequality of people in salaries and wages was one of the serious issues in the departure of the elites from the country. A study showed that poor salaries were one of the factors in nurses’ disappointment with the economic conditions of the country.^31^ Inadequate financial resources to meet family and social responsibilities play a fundamental role in the decision to migrate to improve the living standards of nurses.^26^ A study showed that the great difference in wages was one of the reasons for migration to developed countries.^28^ In a study conducted by Yalma and Asuzu, participants stated that they were driven out of the country due to overwork and low wages.^34^ Vakili and Mobinistated that the difference in wages in professional fields was one of the important factors in migration.^10^ It seems that one of the reasons that prevent the elites from being maintained is the inequality between jobs and different academic fields. Therefore, planning at the macro level should be such that salaries, benefits, and taxes are based on justice and people’s satisfaction. In this way, the elite can be assured that they can show their abilities in fair conditions and away from political issues.

 According to the participants, the uncertain future of children is one of the reasons for the departure of the elites. A study shows that the future security of the family is one of the factors that lead to the departure of skilled human resources.^35^ A study in Pakistan showed that a better opportunity for the future of the family and children had a great impact on the migration of specialists from the country.^36^ Also, Asadi et al showed that children’s safety was an important factor in the migration of Iranian health workers.^37^ It seems that uncertainty about the future of the family and children is an important driver of the departure of elites. Therefore, it is necessary to study various factors affecting this issue and make a comprehensive and effective plan to maintain the elites and their families.

 The existence of social tensions and conflicts is one of the problems of developing countries, which has a great impact on the escape of elites. Studies have shown that socio-political unrest affects personal and family security and is emphasized as a reason for migration.^38^ Sometimes, this is associated with corruption, lack of transparency, lack of accountability, and limited prioritization in health care, which leads to brain drain.^39,40^ Another study reveals that personal freedom is one of the important factors in leaving the country.^35^ According to a study, social issues are one of the factors contributing to migration to developed countries.^36^ Nouri Hekmat et al stated that social factors were one of the factors influencing the migration of Iranian students.^41^ It seems that organizing social issues, dealing appropriately with educated people, and listening to their voices can play an important role in keeping them.

 Lack of meritocracy among managers of organizations is one of the factors that drives the elites out of the country. Studies have shown that lack of meritocracy in appointments has a great impact on elite immigration.^17,42,43^ Fallah Haghighi et al showed that neglecting meritocracy and partisanship in employment led to the migration of skilled people.^44^ Less use of elites in important organizational positions and lack of consultation with them in the affairs of the country are two important factors contributing to dissatisfaction among the elites. It seems that to improve the conditions of the country in all fields, there is a need for expert and elite forces and their use because the factors that determine the progress of a society are its expert and elite forces.

 Changing government policies was one of the most important issues, according to the interviewees. According to the study by Sapkota et al, Nepali nurses were concerned about the country’s political situation and considered it a factor in migration.^45^ According to the study by Okafor and Chimereze, migration of nurses to developed countries was due to weak government policies.^46^ Eissazade et al stated that the first reason for the migration of psychiatric trainees and primary psychiatrists from Iran was political reasons.^47^ Another study showed that political instability was the main pressure factor for migration to other countries.^46^ In other words, many health workers migrate to other parts of the world in search of more stable political conditions.^48^ Another study showed that the Nigerian government did not play a constructive role in the healthcare system, and lack of funds and lack of policies are among the factors contributing to migration.^31^ Atte’s study showed that the relationship between immigration and the role of the government was very important and that the government should investigate why people migrate and what to do to reduce it.^49^ According to a study by Onwujekwe et al, policies and regulations in the healthcare sector are weak and almost nonexistent, thus causing the brain drain in that sector to continue for a long time.^50^ Different government decisions on political issues can affect the fate of elites. It seems that creating specific guidelines in the country legislation is necessary to encourage the elites to stay in the country.

 The results of our study show that the government does not have enough will to solve the problem of brain drain. A study showed that the participants believed that the government lacked accountability and did not understand the problem of brain drain.^31^ One of the reasons for the negligence on the part of politicians is the denial of the issue and lack of attention to the consequences of this human resources crisis. Therefore, it is necessary to dedicate a special program and try to solve the problems of the elites to maintain expert human resources. Also, it seems that understanding the existing conditions and planning to reduce the brain drain are very important issues that must be taken into consideration by developing countries.

 Lack of research opportunities is one of the important factors in the departure of elites. A review study indicates that due to lack of scientific, research, and laboratory facilities in Iran, many elites are thinking of leaving the country.^51^ Studies have shown that poor working conditions, such as shortcomings in infrastructure and necessary equipment are among the reasons for elite migration.^38,39,52^ Another study showed that limited research opportunities were one of the pressure factors in the departure of elites.^52^ It seems that providing the necessary conditions and facilities for various fields, especially laboratory fields that require the most up-to-date devices, can reduce brain drain.

 Predictability of the economy, better quality of life, and high salaries are important factors of attraction for the elite. Ogaboh et al state that good funding and infrastructure in developed countries are part of the motivating factors that draw Nigerian healthcare practitioners to these countries.^53^ According to the study by Kattel and Sapkota, high income, better jobs and work, and higher standards of living are the attractive factors for migration to other countries.^35^ A study showed that better wages and improvements in living conditions were the motives for leaving the country.^49^ Also, a study in Iran showed that access to higher salaries and wages in the destination country was one of the most important driving factors for the migration of skilled workers.^44^ Due to the great depth of this crisis and the reduction of skilled manpower shortly in countries with high immigration statistics such as Iran, government authorities must act faster and reduce this crisis with effective and practical solutions.

 In developed countries, due to the lack of population or specialized personnel, recruiting the elites from other countries is one of the very good options to compensate for this issue. On the other hand, by attracting the elites, the genetic treasury of these countries will increase, and in the long run, it will have a very positive effect on the development of that society. Therefore, the arrival of elite people will be a win-win deal for the destination countries, which can turn them into powerful countries in the near future.

 One of the important factors in the brain drain is economic instability and very high inflation in developing countries like Iran. Due to the conditions of sanctions, the economic mafia at micro and macro levels, and the little meritocracy that exists at the level of organizations, the elites leave the country. Therefore, if the governments are looking for a fundamental change and improvement of the escape of experts in society, there is a need for a coordinated, holistic, and honest approach. The economic problems of society will not be solved by temporary and short-term solutions such as increasing the annual salaries of people because the existence of high inflation will negate all these increases.^48^ Therefore, salary increases should be increased in proportion to inflation, and suitable incentive and support packages should be considered for the elite. Also, establishing international relations with other countries by considering security issues, exchanging science and knowledge with other universities around the world, trying to improve the political and social situation of the country, using elites in key positions, and conveying the sense of being useful and valuable to the elites are important and very effective solutions. Supporting knowledge-based companies and domestic productions and providing job conditions suitable for people’s fields of study are essential to combat brain drain. Therefore, improving the economic, political, and social indicators in the country is one of the main responsibilities of governments. It is necessary to create better job opportunities according to educational and professional qualifications and increase salary packages to improve the economic situation for all members of society, especially the elite.^36^

 Solutions such as brain circulation are popular at the global level. In fact, it is possible to use the knowledge and expertise of people who have migrated (diaspora) for a period of time (about 1 month to a year). This can help to bridge the gap between the immigrant elite and the country of origin.^31^ According to the study by Haghdoost et al, in brain circulation, a person travels between his country and the country of destination. A person comes to the country of origin to work and study and then returns to his country. With this method, the country of origin benefits from the knowledge and experience of the immigrant person.^12^ Darkwa points out that professionals who have migrated bring resources such as financial and human capital to the development of the country of origin.^54^ Brain circulation seems to be achieved only through the joint relationship between the immigrant and the state.^31^ The existence of study opportunities that provide conditions for knowledge exchange between developed and developing countries and specify the years of study is one of the appropriate solutions that can be effective in the short term. According to studies, investing in educational opportunities such as scholarships for research and professional development is helpful.^52,55^ With all the explanations provided, it seems that the basic and important solution for LMICs is to reduce and break the chain of brain drain. Therefore, countries should seek to solve the root of this brain drain to prevent the next crisis, which is the reduction of the elite and expert population and the genetic treasury of developing countries.

 As a strength, this study used the opinions of professors, doctors, and elite immigrants and tried as much as possible to use the maximum diversity in the samples. Difficulties in access to the immigrant elites and the reluctance of some health system experts were some of the limitations of this study.

## Conclusion

 The current study showed that despair about the future, lack of proper respect for the elites, economic instability, high inflation, Inequality of people in salaries and wages, uncertain future of children, existence of social tensions and conflict, lack of meritocracy, changing government policies, little effort by politicians to change the situation, lack of research opportunities, compensation for the lack of population, and predictability of the economy play a big role in brain drain. Also, the proposed solutions from the point of view of health system experts and Iranian elites who emigrated included creating stable economic conditions in the country, creating an atmosphere of hope in the country, increasing respect and dignity for elite students by politicians, using elites in macro-decisions, and creating the job rank for an honorary professor to use the power of the elites. The issue of brain drain is a very important issue that has become more intense in the last decade. The importance of the issue should start from the point where not only elite students and graduates of various medical groups are planning to emigrate, but university professors in the field of health are also leaving the country.Therefore, due to the extent and multi-causes of the issue of brain drain, there is a need for collective thinking and long-term decisions at the macro level of the country. It should be noted that the solutions should aim at reducing the exit of the elites and closing the exit valve because developed countries are constantly creating incentives and support packages for the elites of other countries. Therefore, improving the economic, social, and political situation and providing conditions for the growth and development of the elites should be prioritized by the Iranian authorities.
